# Pathogenesis and Therapeutic Perspectives of Tubular Injury in Diabetic Kidney Disease: An Update

**DOI:** 10.3390/biomedicines13061424

**Published:** 2025-06-10

**Authors:** Jiamian Geng, Sijia Ma, Hui Tang, Chun Zhang

**Affiliations:** 1 Union Hospital, Tongji Medical College, Huazhong University of Science and Technology, Wuhan 430022, China; 2 Department of Nephrology, Union Hospital, Tongji Medical College, Huazhong University of Science and Technology, Wuhan 430022, China

**Keywords:** diabetic kidney disease, tubular injury, mechanism, therapeutics

## Abstract

Diabetic kidney disease (DKD), a well-characterized microvascular complication associated with the progression of diabetes mellitus, has been identified as the leading etiological factor contributing to the global burden of end-stage kidney disease (ESKD). Historically, DKD research has predominantly centered on glomerular mechanisms; however, recent studies have increasingly emphasized the critical role of tubular dysfunction. Extensive evidence has elucidated the key pathological drivers of tubular injury in DKD, encompassing metabolic dysregulation, pro-inflammatory signaling pathways, diverse cellular stress responses, and epithelial–mesenchymal transition (EMT). Furthermore, emerging mechanistic studies reveal that autophagic flux impairment and epigenetic memory formation collaboratively drive cellular senescence in DKD. Regarding the treatment of DKD, various hypoglycemic drugs, as well as hypotensive drugs, and microcirculatory improvers have garnered significant attention. Recently, stem cell-based interventions and precision gene editing techniques have unveiled novel therapeutic paradigms for DKD, fundamentally expanding the treatment arsenal beyond conventional pharmacotherapy. This review synthesizes updated insights into the pathogenesis of tubular injury in DKD and highlights promising therapeutic strategies for managing this condition.

## 1. Introduction

Diabetic kidney disease (DKD), a debilitating microvascular complication of diabetes mellitus (DM), represents a leading cause of chronic kidney disease (CKD) and imposes a substantial burden on global healthcare systems [1,2]. Epidemiological data indicate that approximately 40% of type 2 diabetes mellitus (T2DM) patients and 30% of type 1 diabetes mellitus (T1DM) individuals develop DKD, with the condition accounting for over one-third of end-stage kidney disease (ESKD) cases worldwide [3,4]. Characterized by progressive renal functional decline, DKD severely compromises patient survival and quality of life, underscoring the urgent need for advanced therapeutic interventions.

The pathogenesis of DKD involves multifactorial mechanisms, including glomerular hemodynamic dysregulation, chronic inflammation, fibrotic remodeling, gut microbiome perturbations, and genetic predisposition [5,6,7]. While historical research predominantly emphasized glomerular pathology, marked by hyperfiltration, basement membrane thickening, and podocyte loss [8,9], contemporary studies have identified tubular injury as a critical early event in DKD progression. Emerging evidence suggests that tubular epithelial cell (TEC) dysfunction precedes detectable glomerular damage, manifesting as impaired proliferation, tubular atrophy, interstitial fibrosis, and peritubular capillary loss [10]. Mechanistically, injured TECs drive disease progression through aberrant secretion of reactive oxygen species, profibrotic cytokines, chemokines, and extracellular matrix (ECM) components, creating a self-perpetuating cycle of inflammation and fibrosis [11]. Concurrently, lipid metabolism dysregulation, endoplasmic reticulum stress (ERS), and cellular senescence further exacerbate tubular damage, accelerating the transition to ESKD.

This paradigm shift toward tubular-centric pathogenesis has catalyzed the development of novel therapeutic strategies. Beyond conventional approaches targeting glycemic control, including sodium–glucose transporter 2 (SGLT2) inhibitors and glucagon-like peptide-1 receptor agonists (GLP-1RAs) and blood pressure management like renin–angiotensin system (RAS) inhibitors, emerging interventions address tubular-specific pathways, including redox balance restoration, senescence modulation, and metabolic reprogramming [12]. Current research endeavors focus on dissecting the interplay between tubular injury mechanisms and systemic metabolic derangements, aiming to identify druggable targets for halting DKD progression.

This review synthesizes recent advances in understanding tubular pathophysiology in DKD and evaluates promising therapeutic strategies targeting tubular repair, metabolic homeostasis, and inflammatory–fibrotic cascades. By bridging mechanistic insights with clinical translation, we aim to illuminate pathways for developing precision medicine approaches in DKD management. A systematic search in the electronic databases of PubMed and Google Scholar was made from inception until 31 May 2025, using several MeSH keywords related to DKD, renal tubular injury, etc. Related trials were additionally searched from ClinicalTrials.gov.

## 2. Pathogenesis of Tubular Injury in DKD

### 2.1. Metabolic Dysregulation

**Figure 1:**
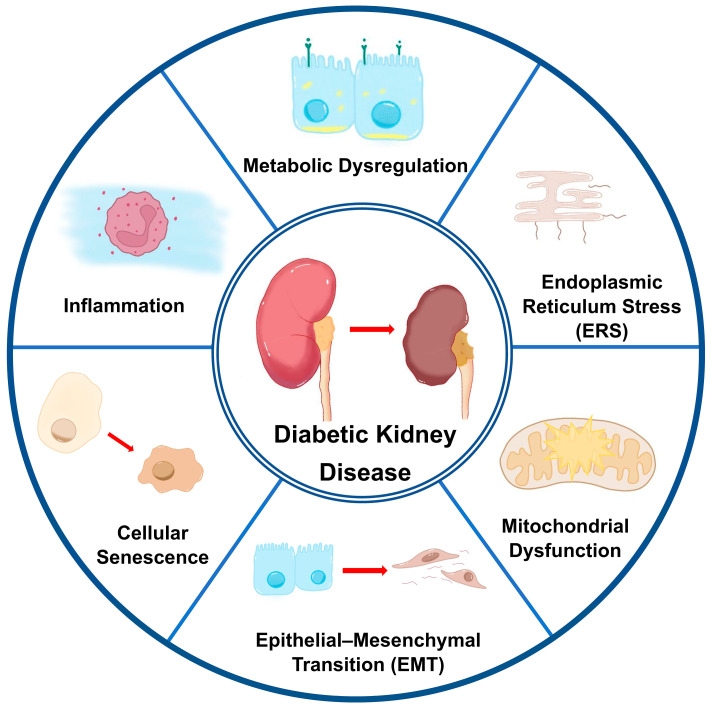
The multifaceted pathophysiological mechanisms of tubular injury in DKD.

As the principal metabolic substrate for biological systems, glucose plays a dual role in the pathogenesis of DKD. Sustained hyperglycemia, a hallmark clinical manifestation of DKD, drives persistent metabolic disturbances through multiple molecular pathways. Pathologically elevated glucose concentrations catalyze the non-enzymatic formation of advanced glycation end products (AGEs) while facilitating intracellular lipid deposition through dysregulated fatty acid (FA) metabolism. These synergistic mechanisms like glycation stress and lipotoxic effects, act as pivotal mediators in the development and progression of renal TEC injury, ultimately driving the progression of DKD [13,14]. The main mechanisms of renal tubular injury are shown in Figure 1.

#### 2.1.1. Hyperglycemia and Advanced Glycation End Products (AGEs)

Hyperglycemia represents the central pathophysiological axis in DKD, functioning as the primordial etiological factor that orchestrates the pathological cascade of tubular injury. Under normal homeostatic conditions, glomerular filtration achieves nearly complete proximal tubular glucose reabsorption via SGLT2 localized on the luminal membrane of early proximal convoluted tubule epithelial cells [15,16]. The residual filtered glucose undergoes secondary reabsorption through SGLT1 transporters predominantly expressed in distal proximal tubular segments, ensuring complete glucose retrieval before the tubular fluid enters the loop of Henle [17].

In diabetic pathophysiology, tubular glucose accumulation arises from mechanisms including glomerular hyperfiltration-driven elevation in filtered glucose load and pathologically augmented reabsorptive capacity mediated by SGLT upregulation. Experimental evidence demonstrates significant membrane density increases in both SGLT isoforms in diabetic rodent models [18,19], a finding corroborated in human studies showing elevated SGLT2 mRNA/protein expression in urinary tubular cells from T2DM patients [20]. This compensatory transporter overexpression establishes a maladaptive positive feedback loop, progressively exacerbating intratubular glucose overload.

Accumulating evidence has established hyperglycemia as the fundamental pathological trigger implicated in the initiation of renal tubular injury in DKD. The pathogenic mechanisms primarily involve two interconnected pathways. Firstly, sustained hyperglycemia coupled with elevated glucose reabsorption induces metabolic dysregulation through mitochondrial dysfunction, as evidenced by diminished mitochondrial reserve capacity in both mesangial and TECs [21]. Furthermore, this energy-intensive reabsorption process exacerbates hypoxia in renal tubules by creating an imbalance between cellular energy demand and oxygen supply [22]. Notably, recent mechanistic studies have revealed that hyperglycemia orchestrates multiple pathological processes, including programmed cell death pathways, activation of stress response systems (ERS/oxidative stress), EMT, and accelerated cellular senescence. Collectively, these findings position hyperglycemia as the central etiological factor driving the progression of tubular injury in DKD.

AGEs, formed through non-enzymatic Maillard reactions between amino groups of proteins/lipids and reducing sugars (glucose, fructose) or reactive carbonyl species (glyoxal) [23,24], constitute critical mediators in DKD pathogenesis. Persistent hyperglycemia in diabetes drives AGE accumulation through dual mechanisms: intracellular overproduction via enhanced glucose reabsorption that elevates substrate availability for endogenous AGE synthesis [25], and impaired renal clearance due to proximal tubular reabsorption of filtered AGEs [26,27]. These accumulated AGEs exert nephrotoxic effects through three principal pathways. Firstly, they induce mitochondrial oxidative stress and pro-inflammatory cytokine activation, initiating tubular epithelial injury [24]. Secondly, AGEs directly stimulate transforming growth factor-β (TGF-β) overexpression in proximal tubular cells through receptor for AGE (RAGE)-dependent signaling [28,29]. Notably, this AGE-TGF-β axis orchestrates pathological ECM deposition by upregulating fibrogenic mediators while downregulating matrix degradation systems. Thirdly, sustained TGF-β activation propels EMT, culminating in progressive tubulointerstitial fibrosis, a hallmark of DKD progression [29].

While AGEs exhibit pleiotropic receptor interactions, their principal pathogenic effects in DKD are mediated through ligand-specific engagement with the RAGE [26,30]. This ligand–receptor axis activates three-tiered signaling cascades, including MAP kinase pathways, Ras-mediated ERK phosphorylation, and JAK/STAT transduction systems [31,32,33]. These convergent pathways drive sustained nuclear translocation of redox-sensitive transcription factors, notably nuclear factor-κB (NF-κB), signal transducer and activator of transcription 3 (STAT3), hypoxia inducible factor-1α (HIF-1α), and activator protein-1 (AP-1), establishing a pro-fibrotic transcriptional landscape.

Mechanistically, the AGE-RAGE interaction executes renal damage through three interconnected mechanisms. Firstly, reactive oxygen species (ROS)-mediated fibrogenesis via nicotinamide adenine dinucleotide phosphate hydrogen (NADPH) oxidase activation generates oxidative stress that perpetuates TGF-β/Smad3 signaling and collagen overproduction [34,35,36]. Secondly, cell cycle subversion through p53/p21-dependent apoptotic pathways induces tubular epithelial loss. Thirdly, senescence programming, evidenced by β-galactosidase activation and telomere attrition, is triggered in proximal tubules both in vivo and in vitro [37].

Notably, these processes synergistically promote tubulointerstitial matrix remodeling characterized by fibrillar collagen (I/III) deposition, basement membrane thickening, and loss of tubular polarity.

#### 2.1.2. Disorders of Lipid Metabolism

Proximal tubular epithelial cells (PTECs) exhibit high energy demands to maintain normal physiological functions, with adenosine triphosphate (ATP) production primarily sustained through mitochondrial free FA β-oxidation [38]. Cellular FA uptake in PTECs is mediated by specialized transport proteins, particularly FA transport protein 2 (FATP2) and CD36 [39,40,41]. Notably, clinical evidence has revealed significant lipid accumulation within PTECs of patients with DKD, a phenomenon strongly associated with disease progression [42]. Advanced lipidomic analyses using desorption electrospray ionization mass spectrometry imaging (DESI-MSI) have identified extensive deposition of diverse lipid species, including free FAs, glycerolipids (GLs), glycerophospholipids (GPs), and sphingolipids (SLs) within cortical proximal tubules of DKD patients [43]. This pathological lipid accumulation induces lipotoxicity through multiple detrimental cellular pathways.

Emerging research delineates several molecular mechanisms underlying lipid metabolism dysregulation in renal tubular injury. Sterol regulatory element-binding proteins (SREBPs), particularly the SREBP-1 isoform, have been identified as master regulators of cellular lipid synthesis and cholesterol metabolism during ectopic lipid deposition (ELD) [44,45]. A novel regulatory axis involving molecular chaperone HSPA8 has been characterized. Genetic ablation of HSPA8 in TECs activates SREBP signaling pathways, while hyperglycemic conditions mimicking DKD pathophysiology reduce HSPA8 expression through NF-κB-mediated transcriptional repression [14]. Concurrently, functional impairment of FATP2 exacerbates PTEC injury by disrupting lipid homeostasis. During fibrotic progression, FATP2 overexpression in PTECs drives pathological remodeling through metabolic reprogramming of lipid pathways, upregulation of pro-fibrotic cytokines, and induction of ERS-mediated apoptosis [46].

The metabolic consequences of incomplete FA oxidation and lipid peroxidation cascade into cellular stress responses. Mitochondrial FA overload induces incomplete β-oxidation, generating excessive ROS that trigger oxidative damage, mitochondrial dysfunction, and subsequent PTEC apoptosis [47,48]. Furthermore, FA metabolites activate inflammatory pathways in TECs, mainly through NF-κB and NOD-like receptor thermal protein domain associated protein 3 (NLRP3) inflammasome signaling, leading to elevated secretion of pro-inflammatory cytokines and amplification of tubular injury [44]. Clinical–pathological correlations demonstrate that lipid deposition intensity positively correlates with necroptosis activation in DKD renal tubules [49].

Lipophagy, a selective autophagic process regulating lipid droplet (LD) turnover, emerges as a critical protective mechanism [50,51,52]. Impaired lipophagic flux in DKD patients results in pathological lipid retention, establishing a direct association between reduced lipophagy, ELD, and lipotoxicity-mediated tubular damage [51]. This deficiency in lipid clearance mechanisms exacerbates the cytotoxic effects of lipid overload.

The pathogenesis of DKD-related renal tubular injury involves multifaceted lipid metabolism dysregulation, encompassing transport abnormalities via FATP2/CD36 dysfunction, synthesis dysregulation through SREBP-1 activation, mitochondrial oxidation defects generating ROS, and impaired catabolic clearance via lipophagy deficiency. These interconnected pathways collectively drive lipotoxic injury, suggesting potential therapeutic targets for mitigating DKD progression.

### 2.2. Inflammation

Emerging evidence substantiates the critical role of inflammatory mechanisms in the pathogenesis and progression of DKD [53]. Hyperglycemic conditions activate several transcription factors regulating pro-inflammatory gene expression, including NF-κB, upstream stimulatory factors (USF1/2), and nuclear factor of activated T-cells (NFAT) [54]. Notably, proteinuria and sustained hyperglycemia synergistically activate NF-κB in TECs, establishing a pro-inflammatory microenvironment that facilitates disease progression through upregulation of inflammatory cytokines [55].

Multiple pathological pathways mediate renal tubular inflammation in DKD. The toll-like receptor (TLR) family, particularly TLR4, functions as a molecular nexus connecting metabolic dysregulation (hyperglycemia/dyslipidemia) with innate immune activation [56,57]. Elevated TLR4 expression in diabetic renal tubules demonstrates a positive correlation with glycated hemoglobin levels and interstitial macrophage infiltration [58]. Mechanistic studies have revealed that miR-630 overexpression in diabetic rodent models significantly downregulates TLR4 expression and reduces pro-inflammatory cytokine levels [59]. Sirtuin 2 (SIRT2), a NAD^+^-dependent deacetylase involved in cellular metabolism, promotes inflammatory responses in diabetic TECs through c-Jun/c-Fos deacetylation. This post-translational modification enhances the transcriptional activity of these AP-1 components, driving downstream pro-inflammatory factor production [60,61,62]. The result indicated the involvement of SIRT2 in the progression of DKD as well as a novel potential therapeutic target. Accumulating studies have demonstrated that proteinuria can stimulate PTECs to produce a series of inflammatory molecules and chemokines [63]. Albumin overload activates Wnt/β-catenin signaling in PTECs, triggering TLR4/NLRP3 inflammasome activation and subsequent chemokine/cytokine production that exacerbate tubulointerstitial inflammation [64]. This pathway establishes a self-perpetuating cycle between glomerular protein leakage and tubular inflammatory responses.

These findings collectively demonstrate that metabolic disturbances, pattern recognition receptor activation, and epigenetic modifications converge to create a pro-inflammatory milieu in renal tubular cells. The resultant cytokine storm and chemokine secretion not only directly damage tubular architecture but also recruit inflammatory infiltrates, ultimately leading to progressive renal fibrosis and functional decline.

### 2.3. Cellular Stress Responses

The adaptive mechanisms of cellular stress responses play a pivotal role in maintaining organismal homeostasis, conferring remarkable resilience against pathological processes and disease progression [65]. In the context of DKD, three primary forms of stress response mechanisms have been implicated in disease pathogenesis and clinical progression. These maladaptive responses manifest as oxidative stress-mediated cellular damage, ERS-induced protein homeostasis disruption, and mitochondrial dysfunction-related metabolic disturbances. Each pathway contributes distinctively to the complex pathophysiology of DKD through interconnected mechanisms of cellular injury and aberrant signaling cascades.

#### 2.3.1. Oxidative Stress

Oxidative stress arises from a pathological imbalance between antioxidant defense mechanisms and ROS production, characterized by excessive accumulation of superoxide anion, hydrogen peroxide, and hydroxyl radicals [66]. These redox-active molecules selectively modify protein function through post-translational alterations in enzymatic activity, subcellular localization, and structural integrity [67]. Under diabetic conditions, chronic hyperglycemia, AGEs, and metabolic dysregulation synergistically drive ROS overproduction that overwhelms endogenous antioxidant systems such as superoxide dismutase and glutathione peroxidase, thereby establishing a self-perpetuating cycle of oxidative damage [68]. This oxidative imbalance accelerates DKD progression by promoting renal tubular injury through inflammation, fibrosis, and apoptosis. Hyperglycemia directly induces mitochondrial ROS (mtROS) overproduction, triggering TEC dysfunction [69], while AGE accumulation in proximal tubules exacerbates oxidative stress via RAGE-mediated NADPH oxidase activation, further amplifying inflammatory cascades [70,71].

Emerging evidence further elucidates the interplay between oxidative stress and signaling pathway dysregulation in DKD pathogenesis. Reduced expression of growth arrest and DNA damage-inducible 45α (GADD45α) in diabetic HK-2 cells disrupts the GADD45α-R-loop axis, exacerbating mitochondrial dysfunction, lipid metabolism impairment, and oxidative injury [72]. Concurrently, hyperactivation of the NF-κB pathway serves as a central mediator of oxidative stress in DKD, with its inhibition demonstrating therapeutic potential to mitigate renal damage [73]. Notably, high glucose-induced upregulation of follistatin-like protein 1 (FSTL1) in HK-2 cells promotes oxidative stress via NF-κB activation, identifying FSTL1 as a novel therapeutic target [74]. Additionally, metabolic reprogramming driven by scavenger receptor CD36 suppresses fatty acid oxidation (FAO) in TECs, inducing a glycolytic shift that enhances mtROS production. This metabolic perturbation activates pro-inflammatory pathways, forming a vicious cycle that perpetuates tubular injury in DKD [75,76].

#### 2.3.2. Endoplasmic Reticulum Stress (ERS)

ERS arises from the accumulation of misfolded proteins within the ER lumen, triggering the unfolded protein response (UPR) to restore proteostasis through coordinated activation of molecular chaperones and degradation pathways [77,78]. In DKD, dysregulated signaling through UPR sensors (IRE1α, PERK, and ATF6) and ER chaperones like GRP78/BiP exacerbates tubular injury by disrupting the autophagy–apoptosis balance and amplifying inflammatory responses [79]. Hyperglycemia-driven activation of the IRE1α/sXBP1 axis mediates NLRP3 inflammasome activation and pyroptosis in renal tubules, while AGEs induce premature senescence of proximal TECs via ERS-dependent signaling cascades [34,80,81]. These pathological processes establish an intricate interplay between ERS and tubular injury mechanisms, including ferroptosis, EMT, and lipid peroxidation [82]. Notably, high glucose conditions activate the XBP1-Hrd1-Nrf2 axis in renal TECs, driving ferroptosis-associated EMT progression through iron-dependent lipid peroxidation [83].

The spatial regulation of ER-mitochondrial crosstalk further modulates DKD pathophysiology, as mitochondria-associated ER membranes (MAMs) coordinate mitochondrial dynamics, autophagic flux, and stress responses [84]. Key regulatory proteins such as protein canopy homolog 2 (CNPY2), an ER-resident UPR promoter, exacerbate tubular damage by disrupting MAM integrity and amplifying ferroptotic cell death [85,86]. Concurrently, defects in protein quality control mechanisms, particularly N-glycosylation processes mediated by enzymes like ectonucleoside triphosphate diphosphohydrolase 5 (ENTPD5), induce UPR activation through impaired folding of N-glycoproteins, culminating in ERS-associated apoptosis [87,88,89]. Intriguingly, compensatory mechanisms emerge during early-stage DKD, where ENTPD5 overexpression alleviates ERS but paradoxically induces pathological cellular hypertrophy through excessive proliferative responses [89]. This delicate balance becomes disrupted in advanced disease stages as chronic hyperglycemia activates the hexosamine biosynthesis pathway (HBP), resulting in elevated UDP-GlcNAc levels that subsequently suppress ENTPD5 expression, thereby creating a maladaptive feedback loop [89].

Emerging therapeutic strategies targeting ERS components, including pharmacological modulators of UPR signaling and glycosylation regulators, demonstrate potential in ameliorating tubular injury [90,91,92]. However, comprehensive elucidation of spatiotemporal ERS regulation in diabetic tubulopathy remains imperative for developing precision interventions against DKD progression.

#### 2.3.3. Mitochondrial Dysfunction

Mitochondrial dysfunction constitutes a central pathogenic mechanism in DKD, particularly within energy-intensive renal tubular cells that exhibit exceptionally high mitochondrial density. These organelles not only serve as central hubs for cellular energy metabolism but also maintain renal tubular homeostasis through dynamic quality control mechanisms. Emerging evidence demonstrates that persistent mitochondrial impairment disrupts both the structural integrity and functional capacity of nephrons, driving DKD progression through multifaceted pathways [38].

Notably, the estrogen-related receptor alpha (ERRα), a nuclear receptor abundantly expressed in proximal tubular cells, emerges as a critical regulator of mitochondrial architecture and bioenergetics [93]. The pathogenic downregulation of ERRα in DKD results from ubiquitin-proteasome-mediated degradation orchestrated by the E3 ligase RBBP6, leading to compromised mitochondrial function in PTECs [94]. Concurrently, mitochondrial quality control (MQC) systems undergo progressive deterioration, exemplified by YAP1 inactivation in renal tubules [95]. This impairment disrupts MQC surveillance mechanisms, triggering aberrant CXCL1 secretion that promotes macrophage polarization, a key inflammatory driver in DKD pathogenesis [96]. Furthermore, the degradation of CaMKKβ via tubular-enriched ligase NEDD4L suppresses adenosine monophosphate-activated protein kinase (AMPK) signaling, effectively disabling a crucial pathway for mitochondrial homeostasis maintenance [97,98].

The metabolic consequences of mitochondrial failure create a vicious cycle through three interrelated mechanisms. First, hyperglycemia-induced tricarboxylic acid cycle overactivation generates excessive reducing equivalents (NADH), culminating in ROS overproduction that preferentially damages mtDNA, particularly vulnerable due to its proximity to the electron transport chain [99,100]. Second, impaired β-oxidation capacity causes toxic lipid accumulation, propagating lipotoxicity-induced cell death and fibrotic remodeling [39,100]. Third, the downregulation of thiosulfate sulfurtransferase (TST) disrupts mitochondrial sulfur metabolism, exacerbating long-chain fatty acid oxidation defects that amplify tubular injury [101]. These intersecting pathways collectively establish the self-perpetuating cascade of oxidative stress, metabolic reprogramming, and inflammatory signaling that characterizes DKD progression.

### 2.4. Epithelial–Mesenchymal Transition (EMT)

EMT represents a fundamental pathological process in DKD, characterized by the dissolution of epithelial cell junctions via E-cadherin suppression and concomitant acquisition of mesenchymal markers such as α-smooth muscle actin (α-SMA), fibroblast-specific protein 1 (FSP1), fibronectin, and vimentin. This phenotypic reprogramming transforms renal TECs into matrix-producing myofibroblasts, driving progressive renal fibrosis through parenchymal architecture destruction and functional decline in tubular reabsorption/secretion [102,103,104].

In DKD, EMT activation is orchestrated by a signaling network involving hyperglycemia-induced metabolic perturbations, inflammatory cascades, and epigenetic dysregulation. Canonical profibrotic pathways, including TGF-β/Smad, MAPK, Wnt/β-catenin, PI3K/Akt, and JAK/STAT, are synergistically activated by cytokines secreted from injured renal cells and infiltrating immune mediators [105,106,107]. Notably, hyperglycemia exacerbates EMT via dual mechanisms, including upregulating tubular basement membrane IgG to amplify TGF-β1-dependent ECM deposition [102,108] and inducing lactate accumulation through metabolic reprogramming. Elevated lactate fuels histone H3K14 lactylation, which recruits the Krüppel-like factor KLF5 to repress CDH1 (E-cadherin) transcription, thereby dismantling epithelial integrity [109,110]. Furthermore, metabolic stress in DKD elevates histone deacetylase 5 (HDAC5), which cooperates with TGF-β signaling to suppress E-cadherin while promoting α-SMA expression. This process is potentiated by m6A RNA hypomethylation due to methyltransferase-like 14 (METTL14) downregulation, which activates PI3K/Akt signaling to sustain HDAC5 overexpression [111]. Furthermore, emerging evidence suggests that EMT can be regulated through multiple signaling pathways beyond conventional mechanisms. Notably, CREB-regulated transcription coactivator 2 (CRTC2), a novel coactivator of cAMP response element-binding protein, has been implicated in glucose metabolism and recently shown to exacerbate EMT progression in diabetic renal tubules through the CREB-Smad2/3 signaling axis [107]. This finding establishes a metabolic–epithelial plasticity link in DKD pathogenesis. Additionally, other pathological contributors to DKD progression have been associated with EMT modulation. Recent investigations reveal that ERS promotes ferroptosis-associated EMT via the XBP1-Hrd1-Nrf2 regulatory cascade in the renal tubular epithelium [83]. These mechanistic insights collectively demonstrate that EMT represents a convergent pathological process that interacts with diverse cellular stress responses, including metabolic dysregulation and organelle stress, ultimately contributing to renal fibrosis in DKD.

The convergence of these pathways culminates in irreversible myofibroblast differentiation, excessive collagen synthesis, and pathological ECM remodeling, hallmarks of tubulointerstitial fibrosis. While emerging therapeutic strategies targeting EMT-related pathways show promise in mitigating fibrosis [112,113], the complexity of crosstalk between metabolic, epigenetic, and signaling networks necessitates further exploration of context-specific molecular hubs for precise intervention in DKD progression.

### 2.5. Cellular Senescence

Cellular senescence, a state of irreversible cell cycle arrest, emerges as a critical pathogenic mechanism in DKD progression. [114,115]. Renal TECs exhibit accelerated senescence under DKD conditions, driven by converging insults including AGEs, oxidative stress, ERS, mitochondrial dysfunction, and chronic inflammation [116]. This senescence program is orchestrated through interconnected molecular cascades [116].

The coagulation–inflammatory axis, mediated by factor XII (FXII), propagates senescence via dual receptor engagement. FXII binding to urokinase-type plasminogen activator receptor (uPAR) activates integrin β1 signaling in TECs, triggering ROS overproduction, DNA damage response, and ultimately senescence-associated secretory phenotype (SASP) activation [117]. Concurrently, metabolic derangements in DKD create a pro-senescence microenvironment. AGEs upregulate the ERS marker GRP78 in renal TECs, which coordinates with p16/p21 signaling to induce premature senescence [81,118]. Lipid overload further exacerbates this process through retinoid X receptor α (RXRα)–mineralocorticoid receptor complexes that directly promote cell cycle arrest [118].

Epigenetic reprogramming adds another layer of regulation. Histone lactylation, a metabolic–epigenetic crosstalk mechanism, drives senescence under hyperglycemic conditions. Counteracting this, the Kruppel-like transcription factor Glis1 binds lactyltransferase KAT5 to suppress histone lactylation, revealing a compensatory anti-senescence pathway [119]. Intriguingly, partial EMT states in TECs establish a senescence-primed niche, where Hedgehog signaling antagonist HHIP amplifies β-galactosidase activity and SASP markers, creating a feedforward loop of fibrotic signaling [120].

These multilayered mechanisms, spanning coagulation signaling, metabolic stress, epigenetic modulation, and partial EMT, converge to sustain SASP-driven inflammation and tissue remodeling. While therapeutic targeting of FXII-uPAR interactions or Glis1-KAT5 epigenetic axes shows potential [119,121,122], the interdependence of senescence drivers in DKD necessitates systems-level approaches to identify nodal regulators for effective intervention.

### 2.6. Gut–Kidney Axis

The Gut–Kidney Axis has emerged as a significant area of research. Recent studies have increasingly focused on alterations in the intestinal environment to elucidate the pathogenesis of various diseases, including kidney diseases [123]. This recognition of crosstalk between the gut environment and the kidney has led to the formulation of the gut–kidney axis hypothesis. Differences in the richness and composition of the gut microbiota have been observed among patients with DKD, patients with DM, and healthy controls [124]. Several studies have identified significant associations between gut microbiota dysbiosis and DKD [125,126]. Furthermore, toxins produced by the gut microbiota can directly damage the kidney and accelerate the progression of kidney disease.

Indoxyl sulfate, a uremic toxin derived from microbial metabolism, contributes to CKD progression by inducing renal tubular injury. It directly promotes apoptotic and necrotic cell death in tubular cells and causes tubulointerstitial injury via oxidative stress, thereby participating in CKD progression [127]. Studies in animal models of diabetic CKD have verified that targeting indoxyl sulfate reduces total cholesterol levels and improves renal function [128]. Similarly, p-cresol, another uremic toxin, can increase renal burden through inflammatory pathways [129]. Research indicates that therapeutic interventions targeting the gut–kidney axis may alleviate DKD progression [130,131]. However, as the existing evidence remains insufficient, the precise mechanisms by which these gut-derived nitrogenous waste products contribute to renal tubular injury in DKD warrant further exploration.

## 3. Therapeutic Perspectives

The management of DKD, a progressive diabetic complication, necessitates sustained pharmacotherapy targeting both metabolic derangements and tubulopathy. Building upon the mechanistic foundation of renal tubular injury outlined in preceding sections, current therapeutic paradigms synergistically address hyperglycemia, hypertension, and specific molecular drivers (Table 1). Conventional glucose-lowering agents and antihypertensive therapies demonstrate nephroprotective efficacy by preserving tubular architecture and mitigating oxidative/inflammatory stress. Emerging frontiers extend beyond metabolic control to innovative modalities. Stem cell-derived extracellular vesicles show potential in tubular regeneration through mitochondrial biogenesis, while CRISPR-based gene editing targets epigenetic regulators like METTL14 to restore m6A homeostasis. These advancements underscore the transition from symptomatic management to mechanism-driven precision medicine in DKD therapeutics.

### 3.1. Hypoglycemic Agents

Hypoglycemic agents exert nephroprotective effects by ameliorating both direct glucotoxic insults and indirect tubular injury through dual mechanisms, including neutralizing primary glucose-mediated cellular damage and intercepting downstream molecular cascades triggered by sustained hyperglycemia.

#### 3.1.1. Metformin

Metformin, a biguanide derivative synthesized from guanidines found in Galega officinalis, is a conventional hypoglycemic agent and a first-line treatment for T2DM [182,183]. Its protective effects against DKD have also been increasingly recognized. One study demonstrated that metformin administration in T2DM patients is associated with a lower risk of overt DKD [184]. Furthermore, patients receiving a combination of metformin and SGLT2 inhibitors exhibit significantly reduced risks of kidney disease progression and mortality compared to those treated with SGLT2 inhibitors alone [185].

Several trials have indicated that metformin can attenuate DKD progression by inhibiting inflammation, oxidative stress, and fibrosis [132]. This protection may be partly achieved by alleviating renal tubular injury. AMPK plays a crucial regulatory role in mitochondrial homeostasis and DKD pathogenesis. As an AMPK agonist, metformin alleviates renal tubulointerstitial fibrosis by activating mitophagy via the AMPK–PINK1–Parkin pathway, thereby reducing mitochondrial damage and ROS generation [133]. Another study confirmed that metformin attenuates renal tubulointerstitial fibrosis by promoting AMPK-induced autophagy and suppressing partial EMT in RTECs, particularly in early-stage DKD [134]. The mammalian target of rapamycin (mTOR), a highly conserved kinase, is implicated in DM, with its overactivation being a critical factor [186]. Existing studies demonstrate that metformin inhibits oxidative stress and apoptosis in DKD by modulating both AMPK and mTOR pathways [132]. Further research is warranted to elucidate metformin’s specific role via the mTOR pathway in protecting renal tubules.

However, the adverse effects of metformin warrant attention. Events such as diarrhea and nausea occur at a higher risk in patients treated with metformin plus lifestyle intervention compared to lifestyle intervention alone [187]. Lactic acidosis, a rare but serious complication, has an incidence of approximately 4.3 cases per 100,000 person-years among metformin users [188]. Future studies should focus on optimizing metformin’s safety profile in DKD treatment, particularly through combination therapies with other agents.

#### 3.1.2. Sodium–Glucose Cotransporter 2 Inhibitors (SGLT2i)

SGLT2i have emerged as multifaceted nephroprotectants in DKD, targeting both systemic hyperglycemia and tubular-specific injury pathways. By inhibiting glucose reabsorption in PTECs, these agents, including dapagliflozin, empagliflozin, and canagliflozin, induce glucosuria while concurrently modulating critical pathomechanisms. SGLT2i have been identified to be beneficial in the treatment of diabetes. Some clinical trials illustrate that the mortality, adverse cardiovascular complications, and acute kidney disease in patients with diabetes are reduced with the utilization of SGLT2i [189,190,191]. The EMPA-KIDNEY trial demonstrated that empagliflozin therapy reduces the risk of kidney disease progression or cardiovascular death in patients with CKD [192]. Similarly, dapagliflozin reduces the risk of a composite outcome comprising a sustained ≥50% decline in estimated glomerular filtration rate (eGFR), end-stage kidney disease, or death from renal or cardiovascular causes in CKD patients, irrespective of DM status [193]. These trials’ results are shown in Table 2.

The role of SGLT2i in metabolism regulation and weight control in diabetes can also lead to the improvement of kidney functions [198]. Animal experiments have demonstrated that treatment with SGLT2i had beneficial effects on lipid metabolism, blood pressure, renal tubular fibrosis, apoptosis, oxidative stress, and inflammation [199]. Several studies also provide evidence of SGLT2i in the protection of DKD, including improvement of kidney functions and inhibition of disease progression [200,201]. Their renoprotective effects stem from coordinated suppression of inflammatory cascades like TNFR1/IL-6/MMP7 downregulation, oxidative stress via AGE-RAGE-ROS axis interruption, and metabolic reprogramming through PKM2-mediated glycolytic flux reduction, collectively attenuating EMT and tubulointerstitial fibrosis [135,136,137].

Mitochondrial protection constitutes another therapeutic axis, where SGLT2i restores MQC by upregulating peroxiredoxin 3 (Prdx3), thereby neutralizing mtROS and preserving tubular bioenergetics [138]. Notably, these agents counteract cellular senescence through HHIP suppression in PTECs and combat novel cell death modalities. Empagliflozin activates AMPK/NRF2 signaling to inhibit ferroptosis, while dapagliflozin reverses lipid peroxidation and mitochondrial dysfunction characteristic of ferroptotic cell death [139,140,141].

The clinical application of SGLT2i continues to be actively investigated. Concurrently, research is exploring the functions of SGLT1, which also mediates glucose and galactose reabsorption. Unlike SGLT2, SGLT1 exhibits a broader tissue distribution, predominantly located in the small intestine but also expressed in the kidney, heart, and brain [202]. SGLT1 is responsible for reabsorbing the majority of dietary glucose in the intestine and the residual filtered glucose in the renal tubule. Interest in SGLT1 inhibitors (SGLT1i) is growing, with some studies suggesting potential benefits for cardiovascular diseases and other conditions [203]. Recent evidence indicates that inhibition of SGLT1 and SGLT2 may offer therapeutic advantages over existing treatments for patients with T2DM, potentially attributable to the renal and cardiovascular protective effects associated with SGLT1 inhibition [204]. To date, SGLT2i remains the primary choice for clinical management of DKD. However, the efficacy of emerging SGLT1/2 inhibitors, which target both transporters with near-equal potency, requires further evaluation. It is noteworthy that adverse events, such as genitourinary infections, have been associated with SGLT2i therapy, necessitating additional research to clarify their associated risks.

Despite these advances, the pleiotropic mechanisms of SGLT2i remain partially deciphered. Current evidence suggests a hierarchical action profile. Primary metabolic effects (glucose-lowering) initiate secondary cellular responses (oxidative stress mitigation), which subsequently modulate tertiary pathophysiological endpoints (fibrosis/senescence). Elucidating spatiotemporal dynamics of these interactions and identifying dominant molecular nodes will be crucial for developing next-generation SGLT2i with enhanced renal tropism and mechanistic precision.

#### 3.1.3. Dipeptidyl Peptidase 4 Inhibitors (DPP-4i)

Glucagon-like peptide-1 (GLP-1), an incretin hormone secreted by intestinal L-cells, plays a pivotal role in glucose homeostasis through potentiation of glucose-dependent insulin secretion from pancreatic β-cells. The biological activity of GLP-1 and gastric inhibitory polypeptide (GIP) is regulated by dipeptidyl peptidase-4 (DPP-4), a membrane-associated protease that rapidly cleaves these incretins [205]. DPP-4i, such as sitagliptin, vildagliptin, and alogliptin, exert therapeutic effects by prolonging the half-life of endogenous incretins, with emerging evidence suggesting additional renoprotective benefits in DKD. Clinical studies have demonstrated that DPP-4i administration improves glycemic control and reduces albuminuria in DKD patients [206]. Vildagliptin has been found to delay the progression of DKD due to the observed renal tissue improvements, and the combination with SGLT2i may have a more therapeutic effect [207].

The effectiveness of DPP-4i in DKD treatment may potentially work through multimodal mechanisms involving anti-inflammatory, antioxidant, and antifibrotic actions [206]. Mechanistically, AGEs induce DPP-4 overexpression in PTECs, creating a pro-inflammatory autocrine loop that can be disrupted by DPP-4 inhibition [142]. Emerging evidence implicates DPP-4 in EMT pathogenesis, with soluble DPP-4 (sDPP-4) shown to activate TGF-β receptor (TGFBR)-dependent EMT signaling in TECs, an effect abrogated by linagliptin treatment [143]. The antifibrotic properties of DPP-4i are further demonstrated through their ability to attenuate TGF-β1-mediated fibrogenesis in PTECs. Specifically, linagliptin disrupts the high glucose-induced interaction between DPP-4 and cation-independent mannose-6-phosphate receptor (CIM6PR) in HK-2 cells, thereby inhibiting TGF-β1 activation and subsequent profibrotic signaling [144]. Furthermore, gemigliptin can downregulate fibrotic gene expression through the inhibition of TGF-β/NF-κB-induced NLRP3 inflammasome activation [145]. DPP-4i also suppress apoptosis by regulating the PI3K/AKT and NF-kB signaling pathways.

In conclusion, DPP-4i play a therapeutic role in renal tubular injury with DKD through the suppression of inflammation, oxidative stress, fibrosis, apoptosis, and so on. However, DPP-4i seem to have a weaker effect compared to other hypoglycemic agents, perhaps due to the fewer studies that focus on DPP-4i. And existing evidence mostly concentrates on combinations with other drugs; thus, further studies are needed to illustrate more functions of DPP-4i.

#### 3.1.4. Glucagon-like Peptide-1 Receptor Agonists (GLP-1RA)

Glucagon-like peptide-1 receptor (GLP-1R) can regulate blood glucose levels and lipid metabolism through specifically binding to the key hormone glucagon-like peptide-1 (GLP-1) [208]. GLP-1RA can imitate the action of endogenous GLP-1 to stimulate GLP-1R, thereby enhancing insulin secretion, inhibiting glucagon release during hyperglycemia, slowing gastric emptying, preventing substantial increases in postprandial glucose, and reducing caloric intake and body weight to achieve glycemic control [146]. Given its functions, GLP-1R and its agonists hold significant therapeutic potential in the treatment of many diseases related to metabolism, such as diabetes.

A study involving 15 kinds of GLP-1RA verified that GLP-1RA can effectively lower hemoglobin A1c (HbA1c) and fasting plasma glucose concentrations, with benefits for weight control in patients with diabetes [209]. GLP-1RA has been demonstrated to reduce the risk of major adverse cardiovascular events (MACEs), all-cause mortality, and worsening kidney function in patients with diabetes [210]. In a major double-blind trial, liraglutide reduced the rate of the first occurrence of death from cardiovascular causes, nonfatal myocardial infarction, or nonfatal stroke among patients with T2DM [194]. Similarly, compared to placebo, semaglutide treatment significantly lowered the risk of a composite of clinically important kidney outcomes and death from cardiovascular causes in patients with T2DM and CKD, further verifying the therapeutic effect of GLP-1RAs in DKD [195].

The protection of GLP-1RA in renal tubules with DKD can be attributed to its inhibition of oxidative stress, inflammation, enhancing renal mitochondrial function, cell death and so on. Given that the dysfunction of mitochondria can induce renal tubular injury, exenatide has been used as a treatment in ameliorating mitochondrial dysfunction, reducing ROS production and apoptosis in renal tubules [147]. Semaglutide can promote the expression of β-klotho (KLB) to mediate ferroptosis inhibition under DKD through the activation of the AMPK signaling pathway, subsequently reducing inflammation and fibrosis as well as alleviation of renal tubular injury [148]. A study verified that liraglutide can suppress lipid synthesis and promote lipolysis to provoke ELD in renal tubules with DKD by promoting AMPK phosphorylation [149]. Similarly, another study found that liraglutide improves renal tubular ELD through modulating gut microbiota composition and increasing serum metabolite 5-OP [150]. GLP-1RA can also enhance AMPK–fatty acid metabolic signaling via macropinocytosis to suppress ferroptosis, contributing to tubular protection [151].

Currently, in the management of diabetes mellitus, the primary deployment of GLP-1RA continues to predominantly involve combination therapy with other pharmacological agents. In a cohort study, the combination of GLP-1RA and SGLT2i was related to a lower risk of MACEs and serious renal events compared to either drug class alone [211]. However, the adverse events associated with GLP-1RAs warrant consideration, as gastrointestinal effects including diarrhea and nausea have been reported during therapy for T2DM [210]. More studies are needed to reveal other mechanisms and functions of GLP-1RA in the treatment of DKD.

### 3.2. Hypotensive Drags

Emerging evidence highlights the therapeutic benefits of hypotensive drugs, demonstrating their efficacy not only in blood pressure regulation but also as critical agents in diabetes management, particularly in addressing the pathophysiological interplay between hypertension and hyperglycemia, two key components of metabolic dysregulation.

#### 3.2.1. Renin–Angiotensin System Inhibitors (RASi)

The renin–angiotensin system (RAS) serves as a cornerstone in preserving renal physiological homeostasis, with accumulating evidence establishing RASi as pivotal therapeutic interventions for DKD.

As pharmacologic agents encompassing angiotensin-converting enzyme inhibitors (ACEi) and angiotensin receptor blockers (ARBs), RASi exhibit the clinically validated capacity to decelerate CKD progression. Their therapeutic efficacy extends beyond hemodynamic modulation through pleiotropic mechanisms involving coordinated suppression of inflammatory cascades, oxidative damage, fibrotic transformation, and programmed cell death pathways. Preclinical investigations revealed that RASi ameliorate proteinuria and tubulointerstitial fibrosis not merely through blood pressure reduction but via molecular interventions such as NF-κB pathway inhibition and attenuation of macrophage infiltration, effectively counteracting inflammatory and fibrotic processes in DKD [152].

Emerging evidence further delineates novel pharmacodynamic mechanisms. Irbesartan demonstrates renal protection through Nrf2/Keap1 pathway activation coupled with NLRP3 inflammasome suppression [153], while losartan exhibits mitochondrial remodeling capabilities in renal proximal tubules, enhancing mitophagy, shifting mitochondrial dynamics toward fusion equilibrium, restoring respiratory competence, and reducing oxidative stress-mediated apoptosis [154]. Concurrently, valsartan orchestrates multi-pathway regulation by targeting genetic networks involved in RAS hyperactivation, AGE-RAGE crosstalk, TGF-β signaling, and PI3K-Akt axis dysregulation, thereby mitigating renal fibrosis and tubular injury [155]. The therapeutic spectrum expands further with olmesartan medoxomil demonstrating autophagy modulation through concurrent inhibition of the AGE/PKC, TLR4/p38-MAPK, and SIRT-1 signaling cascades [156].

This paradigm shift in understanding RASi pharmacology, from traditional antihypertensives to multi-target modulators of cellular degradation, redox balance, and inflammatory signaling, unveils unprecedented therapeutic avenues for DKD management, fundamentally reshaping treatment strategies through molecular precision medicine approaches.

#### 3.2.2. Endothelin Receptor Antagonists (ERAs)

The endothelin (ET) system has emerged as a critical mediator in the pathogenesis of DKD, with endothelin-1 (ET-1) and its receptors (ETA/ETB) exerting multifaceted pathogenic influences through hemodynamic, pro-fibrotic, and inflammatory mechanisms [212]. This complex signaling axis has positioned ERAs as promising therapeutic agents, demonstrating significant nephroprotective potential through antiproteinuric and disease-modifying effects in both preclinical models and clinical trials [213]. Accumulating evidence from randomized controlled studies confirms that ERA monotherapy or combination regimens can substantially reduce albuminuria (38–52% reduction versus placebo) and improve composite renal outcomes in DKD populations, with particular efficacy observed in patients with residual proteinuria despite standard care [214]. The SONAR trial demonstrated that atrasentan reduces the risk of primary composite renal outcomes, including the doubling of serum creatinine and ESKD in patients with DM and CKD, confirming the therapeutic promise of ERAs in DKD [196].

The therapeutic potential of ERA intervention stems from ET-1’s pleiotropic renal actions. Beyond its established role as the most potent endogenous vasoconstrictor, ET-1 activates mitogenic pathways through ETA receptor-mediated stimulation of MAPK and PI3K/Akt cascades, promotes tubular EMT via SMAD-dependent TGF-β signaling, and induces oxidative stress through NADPH oxidase activation [157]. Crucially, ET-1 exerts direct tubular toxicity by disrupting mitochondrial bioenergetics, depressing membrane potential, impairing electron transport chain function, and triggering caspase-dependent apoptosis through ERS pathways [158]. Pharmacological ERA intervention counteracts these pathological processes through targeted receptor blockades. The selective ETA antagonist atrasentan demonstrates mitochondrial protective effects via upregulation of uncoupling protein-2 (UCP2) and superoxide dismutase (SOD2), restoring redox homeostasis while attenuating biomarkers of proximal tubular injury (KIM-1, NGAL) and reducing TEC apoptosis by 67% in diabetic models [159]. Novel ERA derivatives exhibit enhanced therapeutic profiles, with recent in vitro studies showing that ETA/ETB inhibition in high-glucose environments suppresses superoxide overproduction through Nrf2 pathway activation while modulating Bcl-2/Bax balance to prevent HK-2 cell apoptosis [215].

Although ERAs show benefits for DKD treatment, their associated adverse events, particularly fluid retention and anemia, warrant clinical attention [196]. Current therapeutic strategies increasingly employ ERA combination therapies to amplify renoprotection. Synergistic effects have been documented when co-administered with RASi through complementary inhibition of angiotensin II and ET-1 crosstalk while emerging data suggest enhanced glomerular hemodynamic regulation when combined with SGLT2i [216,217,218]. Ongoing phase III trials like SONAR follow-up studies are investigating long-term cardiovascular–renal outcomes of ERA add-on therapy in DKD populations. Future research will focus on developing tissue-specific ERA delivery systems and elucidating the ET system’s role in podocyte–endothelial crosstalk, establishing ET pathway modulation as a cornerstone of emerging precision nephrology approaches for diabetes management.

#### 3.2.3. Mineralocorticoid Receptor Antagonists (MRAs)

The mineralocorticoid receptor (MR), a critical regulator of human physiological processes, demonstrates significant pathophysiological implications when overactivated in diabetes mellitus. Emerging evidence indicates that MR hyperactivation serves as a key driver of oxidative stress, renal inflammation, and fibrotic progression in DKD [219]. Extensive clinical investigations have established that MRAs consistently attenuate albuminuria, slow CKD progression, and inhibit pro-fibrotic/inflammatory pathways in DKD patients [220]. Modern pharmacological classifications distinguish MRAs into steroidal and non-steroidal subtypes, with the latter demonstrating superior therapeutic efficacy through enhanced receptor selectivity and reduced risk of hyperkalemia [221].

Mechanistic studies have revealed that MRAs exert renoprotective effects through multi-target modulation of tubular pathophysiology. These agents preserve proximal tubular integrity by modulation of the mTOR/S6K1 signaling axis, a critical mediator of fibrogenesis, and cellular redox homeostasis [160]. Finerenone, a next-generation non-steroidal MRA, has emerged as a breakthrough therapeutic, demonstrating significant reductions in both renal and cardiovascular morbidity/mortality across diverse DKD populations [161]. Recent molecular investigations elucidate finerenone’s capacity to mitigate oxidative damage, prevent mitochondrial fragmentation, and restore mitophagy through PI3K/Akt/eNOS pathway activation in renal tubular cells [162]. Advanced multi-omics approaches have further identified finerenone-mediated downregulation of ECM production genes and suppression of profibrotic signaling in proximal tubules [163]. Notably, MRAs exhibit novel anti-senescence properties through RXRα/MR pathway inhibition, effectively reducing renal lipid accumulation and interstitial fibrosis in experimental DKD models [118].

MRAs demonstrate therapeutic benefits in DKD. In the FIDELIO-DKD trial, patients with CKD and T2DM receiving finerenone showed reduced risk of both primary outcomes, consisting of kidney failure, a sustained decrease in eGFR, and death from renal causes, and key secondary outcome events compared to the placebo group [197]. Therapeutic optimization strategies emphasize combination therapies, with recent meta-analyses demonstrating enhanced albuminuria reduction and blood pressure control when combining MRAs with SGLT2i [222,223]. This synergistic approach, particularly when employing non-steroidal MRAs, represents a paradigm shift in DKD management by addressing multiple pathogenic pathways simultaneously. The evolving understanding of MRA mechanisms, spanning cellular senescence regulation, metabolic reprogramming, and MQC, continues to expand their therapeutic potential beyond traditional mineralocorticoid antagonism, offering novel avenues for precision medicine in DKD.

### 3.3. Lipid-Modulating Drugs

Emerging evidence underscores the critical role of lipid metabolism dysregulation in the pathogenesis of DKD, with pathological alterations involving triglycerides, cholesterol homeostasis, and lipid droplet dynamics [224]. The underlying mechanisms encompass multifaceted processes, including metabolic reprogramming, ferroptosis, lipophagy dysregulation, and gut microbiota-mediated immunomodulation [225]. These pathophysiological alterations highlight the therapeutic potential of lipid-modulating agents in DKD management.

Clinical investigations have established statins’ significant association with reduced all-cause mortality in CKD populations [164]. Beyond their lipid-lowering properties, statins exhibit pleiotropic renoprotective effects through antioxidant, anti-inflammatory, and anti-fibrotic mechanisms. Experimental studies have revealed that simvastatin ameliorates renal pathology by activating the farnesoid X receptor and Nrf2/HO-1 signaling axis, thereby attenuating oxidative damage, inflammatory responses, and apoptotic pathways in DKD [165]. Similarly, atorvastatin demonstrates renal protective efficacy via suppression of ROS and inhibition of ferroptosis signaling in TECs [166].

Novel therapeutic strategies targeting lipid metabolism show promising results. Fenofibrate administration improves renal function through AMPK/FOXA2/MCAD pathway activation, effectively reducing triglyceride accumulation and tubular apoptosis [167]. GLP-1RAs like liraglutide exhibit dual metabolic benefits by decreasing ELD through modulation of lipogenesis–lipolysis balance in renal tubules [149]. The ANXA1 mimetic peptide Ac2-26 emerges as a potential therapeutic agent, demonstrating improved mitochondrial fatty acid oxidation and reduced lipotoxicity in PTECs, with experimental models showing significant attenuation of tubular injury biomarkers [226]. Furthermore, phytochemical derivatives exhibit renal protective effects through lipid metabolism regulation, suggesting promising avenues for natural product-based interventions [227,228,229].

Notwithstanding these therapeutic advances, emerging evidence cautions against prolonged statin therapy in diabetic models, with studies demonstrating paradoxical exacerbation of insulin resistance, dyslipidemia, and ectopic fat deposition culminating in aggravated renal inflammation and fibrosis [230]. This therapeutic paradox underscores the necessity for rigorous pharmacokinetic studies and large-scale clinical trials to establish optimal dosing regimens and evaluate long-term safety profiles.

The evolving landscape of lipid-targeted therapies presents opportunities and challenges in DKD management. Future research should prioritize defining tissue-specific actions of lipid modulators, developing combination therapies targeting multiple pathogenic pathways, and establishing personalized treatment algorithms grounded in individual metabolic profiles.

### 3.4. Stem Cell Therapy

Emerging as a groundbreaking therapeutic frontier in DKD management, stem cell therapy demonstrates multifaceted renoprotective effects through diverse cellular mechanisms. Current translational approaches utilize stem cells derived from bone marrow (BM), umbilical cord/amniotic fluid (UC/AF), urinary sources, and adipose tissue, all showing efficacy in ameliorating renal dysfunction by reducing proteinuria, oxidative stress, EMT, and fibroinflammatory processes [231]. Among various stem cell types, mesenchymal stem cells (MSCs) have emerged as the most clinically promising modality, exerting therapeutic effects through immunomodulation, MQC enhancement, autophagy restoration, and anti-fibrotic mechanisms [232]. Clinical validation comes from a randomized trial demonstrating the acceptable safety profile of bone marrow-derived ORBCEL-M cells in DKD patients, despite observed transient adverse effects [233].

Mechanistically, MSCs mediate renal protection through sophisticated cellular crosstalk. Human MSCs engage in paracrine interactions with PTECs, effectively suppressing chronic inflammatory signaling cascades [168]. These effects are partially mediated by MSC-derived small extracellular vesicles (sEVs) carrying regulatory microRNAs, notably miR-23a-3p, which attenuates renal fibrosis and inflammation through KLF3/STAT3 pathway modulation in hyperglycemic HK-2 cells [169]. Bone marrow MSCs (BMSCs) demonstrate therapeutic actions in hyperglycemic environments by mitigating renal inflammation/fibrosis while inhibiting ferroptosis via MAPK signaling suppression [170]. Complementary mechanisms include mitochondrial transfer to PTECs, which reduces apoptosis and ROS production through MQC restoration [171,172]. Umbilical cord-derived MSCs (UC-MSCs) exhibit distinct anti-fibrotic properties via exosome-mediated Hedgehog/SMO pathway inhibition, effectively blocking EMT progression [173]. Furthermore, MSC-derived exosomes counteract autophagy impairment in DKD by modulating mTOR-dependent signaling pathways [174].

The therapeutic landscape extends to induced pluripotent stem cells (iPSCs), reprogrammed somatic cells with multilineage differentiation capacity. Their potential to generate renal tubular progenitor cells offers novel opportunities for targeted tubular regeneration in DKD [234]. While autologous stem cell sources theoretically mitigate immunological rejection risks, current clinical translation remains constrained by methodological challenges. Most evidence remains predominantly confined to preclinical investigations, necessitating rigorous clinical trials to establish standardized protocols, optimize delivery methods, and validate long-term therapeutic outcomes across diverse DKD populations.

### 3.5. Gene Therapy

Accumulating evidence highlights the pivotal role of genetic and epigenetic dysregulation in the pathogenesis of DKD, with disease progression driven by aberrant gene expression mediated through canonical signaling pathways, transcription factors, and epigenetic modifications. Notably, chromatin remodeling, DNA methylation, and non-coding RNA (ncRNA)-mediated regulation have emerged as critical mechanisms underlying renal pathology [235]. These discoveries position gene therapy as a promising strategy for targeting disease-specific molecular alterations in DKD.

Epigenetic reprogramming has been implicated in renal fibrosis, a hallmark of DKD progression. Meta-analyses reveal distinct epigenetic signatures in renal tubular cells during fibrotic transformation, including dysregulated DNA methylation patterns and histone modifications [236]. The RNA methyltransferase METTL3, a key regulator of N6-methyladenosine (m6A) modification, promotes EMT in TECs by enhancing mRNA stability and the translational efficiency of ZEB2, a master EMT inducer. Pharmacological inhibition of METTL3 or targeted m6A demethylation of ZEB2 mRNA significantly attenuates EMT and fibrosis in experimental models [175,176]. Concurrently, histone methylation dynamics regulate fibrogenic gene expression, with therapeutic modulation of histone-modifying enzymes demonstrating efficacy in reducing tubular injury and ECM deposition [177].

The regulatory potential of ncRNAs in DKD pathophysiology has garnered substantial attention. Multiple microRNAs (miRNAs) function as endogenous suppressors of the TGF-β1/SMAD pathway, a central driver of renal inflammation and fibrosis. For instance, miR-30c and miR-141 mitigate EMT in TECs by antagonizing TGF-β1 signaling, while miR-122-5p supplementation alleviates tubular injury and interstitial fibrosis in preclinical DKD models [178,179,180]. Mechanistically, these miRNAs directly target SMAD7, a negative regulator of TGF-β1 signaling, restoring its expression to counteract fibrotic progression [181]. Emerging therapeutic strategies leverage miRNA mimics or nanoparticle-based delivery systems to enhance endogenous miRNA activity, offering precision in modulating pathogenic pathways.

While gene therapy holds transformative potential through its capacity for precise molecular targeting and upstream pathway modulation, the inherent complexity of genetic networks presents significant challenges. The pleiotropic effects of epigenetic modifiers and ncRNAs necessitate meticulous identification of nodal regulatory points within interconnected signaling cascades. Current research priorities include optimizing tissue-specific delivery systems, resolving off-target effects of epigenetic interventions, and elucidating crosstalk between genetic and metabolic pathways in DKD. This evolving paradigm underscores the necessity for multidimensional approaches integrating gene editing technologies, epigenetic modulators, and ncRNA-based therapeutics. Future advancements in single-cell sequencing and CRISPR-based screening platforms may enable the development of personalized gene therapy regimens tailored to individual epigenetic landscapes in DKD progression.

## 4. Conclusions

Given the critical involvement of renal tubular injury in exacerbating DKD, this review synthesizes key mechanistic pathways, including metabolic dysregulation, inflammatory activation, cellular stress responses, EMT, and senescence pathways, collectively forming an interconnected pathological network driving disease progression. Current therapeutic advancements targeting tubular pathophysiology span multiple modalities, exhibiting renoprotective efficacy. Emerging strategies, including regenerative medicine approaches like mesenchymal stem cell therapy and CRISPR-based gene editing, further underscore the expanding therapeutic armamentarium. Despite these advances, unresolved mechanistic complexities necessitate future investigations to delineate novel molecular drivers of tubular injury and validate targeted interventions through translational studies. Integrating multi-omics profiling, advanced disease modeling, and clinical trials will be pivotal in advancing precision therapeutics for DKD.

## Figures and Tables

**Table 1:** Therapies targeting renal tubule injury in the management of DKD.

Therapeutic Class	Drug Example	Protective Mechanisms	References
Hypoglycemic Agents	Metformin	Activate mitophagy, reduce mitochondrial damage and ROS generation (AMPK-PINK1-Parkin pathway), and inhibit oxidative stress and apoptosis (AMPK and mTOR pathway)	[132,133,134]
	SGLT2 Inhibitors (e.g., empagliflozin, dapagliflozin, canagliflozin)	Reduce glucose reabsorption, suppress inflammation (TNFR1/IL-6/MMP7), inhibit oxidative stress (AGE-RAGE-ROS), modulate metabolism (PKM2), protect mitochondria (Prdx3), inhibit ferroptosis (AMPK/NRF2), and counteract senescence (HHIP suppression)	[135,136,137,138,139,140,141]
	DPP-4 Inhibitors (e.g., sitagliptin, vildagliptin, alogliptin)	Inhibit DPP-4 activity, suppress inflammation (AGE-DPP4 axis), inhibit EMT (TGFBR), attenuate fibrosis (TGF-β1 signaling), and reduce apoptosis (PI3K/AKT, NF-κB)	[142,143,144,145]
	GLP-1R Agonists (e.g., exenatide, semaglutide, liraglutide)	Enhance insulin secretion, inhibit oxidative stress, reduce inflammation, improve mitochondrial function, inhibit ferroptosis (AMPK pathway), and regulate lipid metabolism	[146,147,148,149,150,151]
Hypotensive Drugs	RAS Inhibitors (e.g., ACEi, ARBs like irbesartan, losartan, valsartan, olmesartan)	Inhibit NF-κB, reduce inflammation and fibrosis, activate Nrf2/Keap1, suppress NLRP3 inflammasome, remodel mitochondria, and modulate AGE-RAGE-TGFβ-PI3K pathways	[152,153,154,155,156]
	Endothelin Receptor Antagonists (e.g., atrasentan)	Block ET-1 signaling, reduce oxidative stress (NADPH oxidase inhibition), suppress EMT (TGF-β-SMAD), and protect mitochondria (UCP2, SOD2 upregulation)	[157,158,159]
	Mineralocorticoid Receptor Antagonists (e.g., finerenone)	Inhibit mTOR/S6K1, activate PI3K/Akt/eNOS, improve mitophagy, reduce oxidative stress and fibrosis, and counteract senescence (RXRα/MR pathway)	[118,160,161,162,163]
Lipid-Modulating Drugs	Statins (e.g., simvastatin, atorvastatin), Fenofibrate	Reduce oxidative stress, inflammation, and ferroptosis; modulate lipid metabolism via AMPK/FOXA2/MCAD pathways	[164,165,166,167]
Stem Cell Therapy	Mesenchymal Stem Cells (MSCs)	Reduce inflammation, fibrosis, and apoptosis, transfer mitochondria, restore autophagy, and modulate KLF3/STAT3 pathways	[168,169,170,171,172,173,174]
Gene Therapy	METTL3 Inhibitors, miRNAs (e.g., miR-30c, miR-141, miR-122-5p)	Modify epigenetic regulation (m6A methylation), inhibit EMT/fibrosis, restore SMAD7, and suppress TGF-β1 pathways	[175,176,177,178,179,180,181]

SGLT2, sodium–glucose transporter 2; DPP-4, dipeptidyl peptidase-4; GLP-1R, glucagon-like peptide-1 receptor; RAS, renin–angiotensin system; METTL3, methyltransferase-like 3.

**Table 2:** Recent clinical trials related to DKD or DM.

Drugs	Clinical Trials	Study Design	Number of Patients	Primary Outcomes	Secondary Outcomes	Reference
Empagliflozin	EMPA-KIDNEY study	Randomized, double-blind, placebo-controlled trial in CKD patients with or without diabetes; empagliflozin 10 mg daily; median follow-up 2.0 years.	6609	28% reduction in progression of kidney disease or CV death (HR 0.72, *p* < 0.001).	Lower all-cause hospitalization; no significant effect on CV death or HF hospitalization.	[192]
Dapagliflozin	DAPA-CKD study	Randomized, placebo-controlled trial in CKD patients with or without T2D; dapagliflozin 10 mg daily; median follow-up 2.4 years.	4304	39% reduction in sustained GFR decline, kidney failure, or renal/CV death (HR 0.61, *p* < 0.001).	29% reduction in CV death or HF hospitalization; 31% reduction in all-cause mortality.	[193]
Liraglutide	LEADER study	Randomized, double-blind trial in T2D with high CV risk; liraglutide 1.8 mg (or the maximum tolerated dose) daily; median follow-up 3.8 years.	9340	13% reduction in CV death, nonfatal MI, or stroke (HR 0.87, *p* = 0.01).	22% reduction in CV death; 15% reduction in all-cause mortality; fewer MI and stroke.	[194]
Semaglutide	FLOW study	Multicenter, randomized, double-blind, placebo-controlled trial in patients with T2D and CKD; semaglutide 1.0 mg weekly; median follow-up 3.4 years.	3533	24% reduction in major kidney disease events (HR 0.76, *p* = 0.0003).	Slower eGFR decline; 18% reduction in MACE; 20% reduction in all-cause mortality; fewer serious adverse events.	[195]
Atrasentan	SONAR study	Double-blind, placebo-controlled trial in CKD with T2D; atrasentan 0.75 mg daily; median follow-up 2.2 years.	2648	35% reduction in doubling serum creatinine or kidney failure (HR 0.65, *p* = 0.0047).	No significant difference in CV death or HF hospitalization.	[196]
Finerenone	FIDELIO-DKD study	Randomized, double-blind trial in CKD with T2D; maximum dose on the manufacturer’s label; median follow-up 2.6 years.	5734	18% reduction in kidney failure, GFR decline, or renal death (HR 0.82, *p* = 0.001).	14% reduction in CV events (HR 0.86, *p* = 0.03); higher discontinuation due to hyperkalemia.	[197]

CV death, cardiovascular death.

## Data Availability

Not applicable.
